# Clinical Impact of Biofilm-Producing Carbapenem-Resistant Acinetobacter baumannii: Diagnosis and Treatment Challenges

**DOI:** 10.7759/cureus.110019

**Published:** 2026-06-01

**Authors:** Devika G Bhati, Harsha V Patil, Satish R Patil

**Affiliations:** 1 Microbiology, Krishna Institute of Medical Sciences, Krishna Vishwa Vidyapeeth (Deemed to be University), Karad, IND

**Keywords:** acinetobacter baumannii, antimicrobial resistance, biofilm, carbapenem resistance, crab, nosocomial infections

## Abstract

Carbapenem-resistant *Acinetobacter baumannii* (CRAB) has emerged as a major nosocomial pathogen associated with significant morbidity and mortality, particularly in intensive care unit (ICU) settings. Its remarkable ability to survive in adverse environments, persist on medical devices, and rapidly acquire multidrug resistance has made it a critical global healthcare concern. This review aims to provide a comprehensive overview of the epidemiology, risk factors, antimicrobial resistance mechanisms, and pathogenicity of CRAB, with a special emphasis on the role of biofilm formation. CRAB infections are strongly associated with prolonged hospitalization, mechanical ventilation, previous antibiotic exposure, and invasive procedures. The organism exhibits multiple resistance mechanisms, including carbapenemase production, efflux pumps, porin modifications, and horizontal gene transfer, which significantly limit therapeutic options. A key virulence factor is its capacity to form biofilms on biotic and abiotic surfaces, enhancing bacterial survival, immune evasion, and resistance to antimicrobial agents. Biofilm-associated infections are often chronic, recurrent, and difficult to eradicate, particularly in device-related infections. The interplay between biofilm formation and antimicrobial resistance further complicates treatment outcomes. Current management strategies rely on last-resort antibiotics, combination therapy, antimicrobial stewardship, and strict infection control practices, while emerging therapies targeting biofilms offer promising alternatives. Understanding these complex mechanisms is essential for developing effective therapeutic and preventive strategies against CRAB infections.

## Introduction and background

The ability of carbapenem-resistant *Acinetobacter baumannii *(CRAB) to survive in hostile environments and rapidly acquire resistance to multiple antimicrobial agents has made it a major pathogen in modern healthcare settings. *A. baumannii *is a gram-negative, aerobic, non-fermentative coccobacillus that has emerged as an important opportunistic pathogen associated with healthcare-associated infections, particularly in intensive care units (ICUs). *A. baumannii *infections have increased globally over the last decade, especially in hospital settings, where they are associated with severe clinical outcomes [1]. The bacterium is well known for its ability to persist on biotic and abiotic surfaces, including medical equipment, facilitating its transmission within healthcare facilities.

Current treatment strategies for multidrug-resistant (MDR) *A. baumannii* infections include carbapenems, sulbactam-based therapy, polymyxins (colistin or polymyxin B), tigecycline, minocycline, and combination regimens depending on antimicrobial susceptibility patterns. However, the increasing prevalence of carbapenem resistance has substantially limited effective therapeutic options.

Because carbapenems are frequently regarded as last-line antibiotics for treating serious infections, the global increase in carbapenem resistance in *A. baumannii* is particularly alarming. The development of CRAB restricts available treatments, increases treatment failure rates, lengthens hospital stays, and raises mortality [2]. Because the bacterium can cause potentially fatal infections, such as ventilator-associated pneumonia (VAP), bloodstream infections, and wound infections, this issue is especially noticeable in critically sick patients. *A. baumannii* is commonly isolated from patients in critical care units (ICUs) who have been hospitalized for a long time, are on mechanical ventilation, or have previously been exposed to antibiotics. Resistant strains are more likely to colonize and infect the environment created by these risk factors [3].

This bacterium poses a significant challenge to infection control systems because of its capacity to adapt and endure under selection pressure. CRAB’s ability to produce biofilms is another important component in its success. Biofilms are organized groups of bacteria that are shielded from human immune reactions and antibiotics by a matrix that they create on their own. This characteristic is important for recurrent and chronic infections, in addition to improving bacterial survival.

To effectively manage the spread of CRAB and enhance patient outcomes, it is crucial to comprehend its epidemiology, resistance mechanisms, and pathogenic aspects. With a focus on the role of biofilm development in antimicrobial resistance, this article seeks to give a thorough overview of these factors.

Methodology

Research Strategy

**Figure 1:**
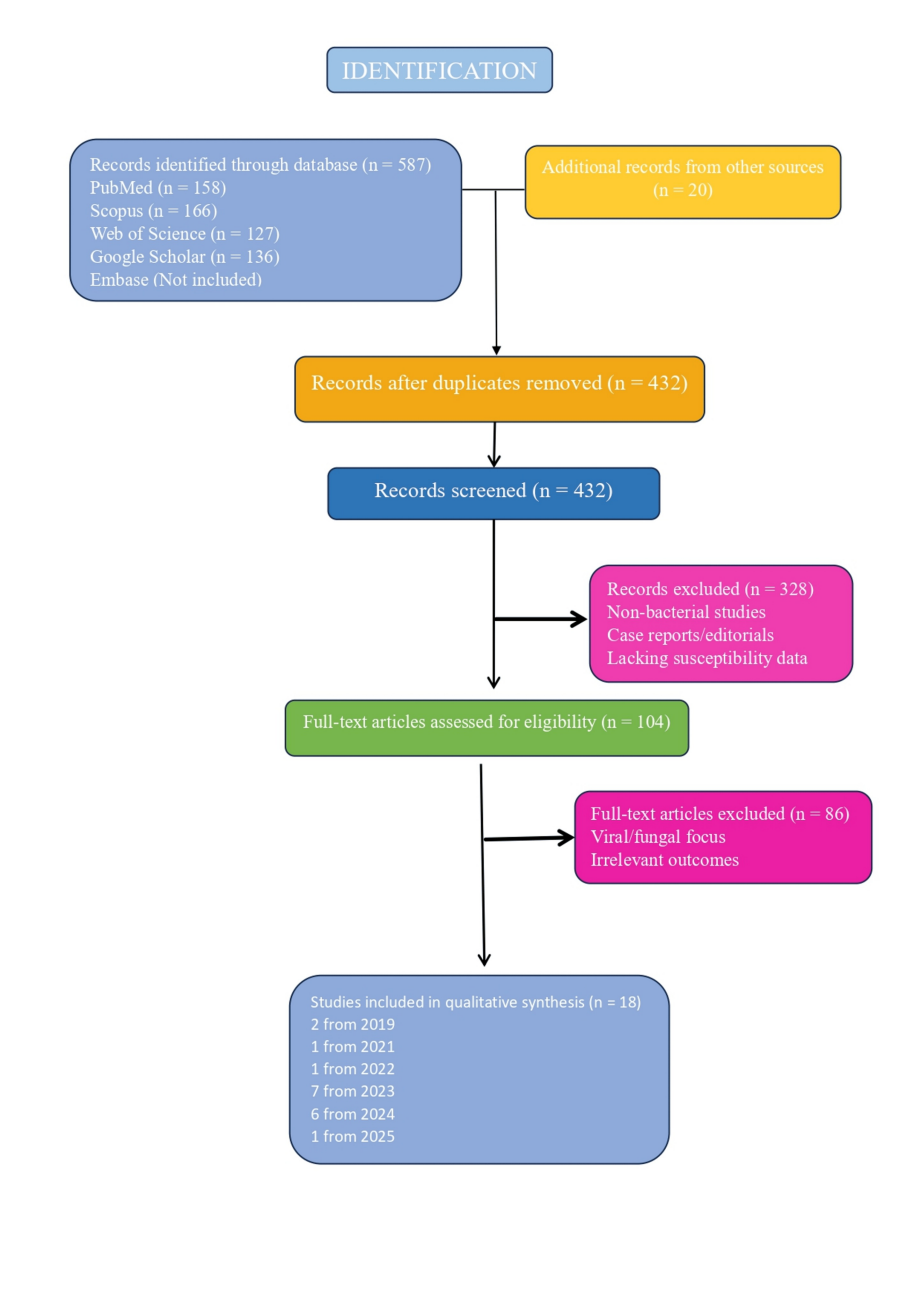
Article selection and screening process. Canva was used for figure formatting/layout purposes; the figure was not created using Canva AI or any AI-generated feature. n=number of articles

The literature search included studies published between 2019 and 2025, focusing on the epidemiology, biofilm formation, virulence mechanisms, antimicrobial resistance, and treatment strategies of CRAB. Only English-language studies relevant to *A. baumannii* in healthcare and ICU settings were included. Eligible studies comprised original research articles, review articles, and clinical studies reporting microbiological, molecular, and antimicrobial susceptibility data. Studies unrelated to *A. baumannii*, lacking resistance or susceptibility data, or consisting of case reports, editorials, conference abstracts, duplicate publications, and non-relevant studies were excluded. Search strategies were customized according to the indexing format of each database using Boolean operators (AND, OR) to optimize retrieval. In PubMed, the search strategy used was "*Acinetobacter baumannii*" OR "CRAB" AND "biofilm formation" OR "virulence mechanisms" OR "antimicrobial resistance" OR "treatment challenges." In Scopus, the search was conducted using TITLE-ABS-KEY ("*Acinetobacter baumannii*" OR "CRAB") AND TITLE-ABS-KEY ("biofilm formation" OR "virulence mechanisms" OR "antimicrobial resistance" OR "treatment challenges"), whereas Web of Science used TS=("*Acinetobacter baumannii*" OR "CRAB") AND TS=("biofilm formation" OR "virulence mechanisms" OR "antimicrobial resistance" OR "treatment challenges"). Google Scholar was searched using the keywords "*Acinetobacter baumannii*" OR "CRAB" AND "biofilm formation" OR "virulence mechanisms" OR "antimicrobial resistance" OR "treatment challenges". After retrieval, duplicate studies were removed, and the remaining records underwent title and abstract screening followed by full-text eligibility assessment according to predefined inclusion and exclusion criteria. Following screening and eligibility assessment, 18 studies published between 2019 and 2025 were included in the final qualitative synthesis. The article selection and screening process is illustrated in Figure 1.

## Review

Epidemiology and risk factors of CRAB

Over the last 10 years, there has been a notable rise in the prevalence of CRAB, especially in hospital and ICU settings. It is widely acknowledged that this organism is one of the main global sources of infections linked to healthcare, particularly in low- and middle-income nations [1]. The organism's capacity to endure in-hospital settings, widespread antibiotic usage, and inadequate infection control procedures are the primary causes of the rising incidence.

Due to the presence of critically sick patients and invasive procedures, *A. baumannii *infections are more commonly documented in ICUs worldwide than in normal wards. Research has indicated that CRAB is frequently linked to surgical site infections, bloodstream infections, urinary tract infections, and VAP [3].

The organism spreads more easily because of its persistence on surfaces such as ventilators, catheters, and medical equipment. The burden of CRAB infections increased rapidly between 2020 and 2025, according to recent epidemiological statistics. The overuse of broad-spectrum antibiotics, especially carbapenems, which strongly select for resistant bacteria, is directly associated with the increase in resistance rates [2].

A number of risk factors for CRAB infections have been found, particularly in ICUs. Prolonged hospital stays, mechanical ventilation, antibiotic exposure in the past, invasive surgeries, and underlying comorbidities such as diabetes or immunosuppression are some of these [4]. Because they significantly increase the risk of colonization and infection, mechanical ventilation and extended ICU stays are thought to be the most important causes. Because of these epidemiological variables, CRAB poses a significant threat to healthcare systems, necessitating stringent “infection control procedures” and antimicrobial stewardship initiatives to stop its spread (Tables 1-3).

**Table 1:** Comprehensive Summary of Included Studies on CRAB [1-17] This table summarizes the characteristics and major findings of the 17 studies included in this review article. It highlights study design, objectives, key findings, resistance mechanisms, biofilm-related observations, and clinical significance associated with carbapenem-resistant* Acinetobacter baumannii* (CRAB). MDR, multidrug-resistant; ICU, intensive care unit; VAP, ventilator-associated pneumonia; OXA, oxacillinase

Author	Year	Study type	Study objective	Key findings	Biofilm/resistance findings	Clinical significance
Müller et al.	2023	Review	Global epidemiology of CRAB	Rising worldwide prevalence	Biofilm contributes to persistence and resistance	Increased morbidity and mortality
Thacharodi et al.	2024	Review	Emerging antibiotic regimens	Increasing resistance trends	Carbapenemase-mediated resistance associated with biofilms	Necessity for novel therapeutic strategies
Cavallo et al.	2023	Review	CRAB infections in critically ill patients	High ICU prevalence	Strong biofilm-related persistence	Severe ICU-associated infections
Huang et al.	2019	Clinical study	VAP treatment outcomes	CRAB common in ventilated patients	Device-associated biofilm formation	Increased morbidity and mortality
Wu et al.	2023	Review	Molecular resistance mechanisms	Multiple resistance pathways identified	Efflux pumps and carbapenemases associated with persistence	Limited treatment options
Mendes et al.	2023	Review	Resistance and biofilm co-regulation	Strong interaction between resistance and biofilm	Enhanced survival and gene transfer	Increased pathogenicity
Naseef et al.	2024	Review	Biofilm development	Detailed stages of biofilm formation	Mature biofilm increases resistance	Persistent chronic infections
Upmanyu et al.	2022	Review	Factors affecting biofilm formation	Environmental stress promotes biofilm	Stress-induced adaptive resistance	Potential therapeutic targets
Smitran et al.	2023	Experimental study	Biofilm eradication strategies	Selenium nanoparticles showed activity	Biofilm inhibition observed	Potential novel anti-biofilm therapeutic approach
Gedefie et al.	2023	Meta-analysis	Prevalence of biofilm-producing isolates	High prevalence of biofilm producers	Strong association with MDR strains	Greater clinical burden
Vintilă et al.	2024	Comparative study	Biofilm and resistance comparison	Resistant strains form stronger biofilms	Carbapenemase-producing isolates persistent	Increased recurrence
Choudhary and Shariff	2025	Laboratory study	Characterization of CRAB isolates	*OXA* genes are frequently identified	Strong biofilm-forming isolates are common	Important for diagnosis and epidemiological surveillance
Iovleva et al.	2024	Review	Treatment approaches	Combination therapy beneficial	Biofilm reduces antibiotic penetration	Improved treatment guidance
Rajangam and Narasimhan	2024	Review	Anti-biofilm strategies	Novel virulence-targeted therapies discussed	Biofilm inhibition improves susceptibility	Emerging therapeutic approaches
Mohamed et al.	2023	Review	Biofilm therapeutic targets	Multiple virulence factors identified	Biofilm central to pathogenesis	Potential drug targets
Yadav et al.	2024	Experimental study	Biofilm inhibition methods	Reduced biofilm formation was observed	MDR isolates associated with biofilm	Potential clinical application
Gedefie et al.	2021	Review	Biofilm role in disease pathogenesis	Biofilm important in chronic infection	Enhanced adhesion and persistence	Recurrent infections

**Table 2:** Year-Wise Comparison of CRAB Epidemiology This table presents a year-wise comparison of the epidemiological trends of carbapenem-resistant *Acinetobacter baumannii *(CRAB) from 2020 to 2025. It highlights key findings, including the emergence and progression of antimicrobial resistance, increasing biofilm-associated infections, and the growing prevalence of CRAB in intensive care units (ICUs). The table also outlines the corresponding clinical impact, such as increased infection severity, persistence, and association with risk factors such as mechanical ventilation. The information is summarized based on the findings from the cited references.

Year	Key findings	Clinical impact	References
2020-2021	Moderate rise in CRAB cases, emerging resistance patterns	Initial increase in ICU infections	[1]
2022	Increased biofilm-related infections and resistance mechanisms	More persistent and chronic infections	[2]
2023	High ICU prevalence, association with ventilator use	Associated with poor clinical outcomes and increased ICU morbidity	[3]
2024-2025	Significant global spread, strong risk factor association	Significant global spread, strong risk factor association	[4]

**Table 3:** Major Risk Factors for CRAB Infection This table summarizes the major risk factors associated with carbapenem-resistant *Acinetobacter baumannii* (CRAB) infections, along with their underlying explanations and clinical significance. Key factors such as prolonged ICU stay, mechanical ventilation, previous antibiotic exposure, use of invasive devices, and contaminated hospital environments contribute to increased colonization, infection risk, and transmission of multidrug-resistant organisms. The data are compiled from referenced studies.

Risk factor	Explanation	Clinical significance	References
Prolonged ICU stay	Longer exposure to hospital pathogens	Increases colonization risk	[4]
Mechanical ventilation	Direct airway access	Major cause of VAP	[4]
Previous antibiotic use	Selects resistant strains	Leads to MDR organisms	[4]
Invasive devices	Catheters, ventilators	Entry point for infection	[4]
Hospital environment	Contaminated surfaces	Facilitates transmission	[4]

Mechanisms of antimicrobial resistance in *Acinetobacter baumannii*

One of the primary factors contributing to*A. baumannii's *clinical significance is its capacity to acquire resistance against several types of antibiotics. This bacterium has developed a number of strategies over time that enable it to endure the presence of strong antibiotics such as carbapenems. Treatment becomes more challenging and therapeutic alternatives are limited when these resistance mechanisms co-exist [5].

The synthesis of enzymes called carbapenemases is one of the most significant resistance mechanisms. Before carbapenem drugs can affect the bacterial cell, these enzymes degrade them. Carbapenem resistance is mostly caused by oxacillinase (OXA)-type β-lactamases, which are frequently present in*A. baumannii. *Moreover, resistance levels may be further increased by the presence of metallo-β-lactamases (MBLs) [5].

Modifications to the bacterial outer membrane, typically porin channels, allow antibiotics to enter bacterial cells. However, these porins can be altered or reduced by* A. baumannii,* which reduces drug access. Because of this, even potent antibiotics are unable to get to their intended locations within the cell. Another important factor contributing to resistance is efflux pumps. These protein systems aggressively remove antibiotics from the bacterial cell. Antibiotics are less effective because the bacterium keeps its intracellular concentration low by constantly eliminating the medication. MDR bacteria are more vulnerable to efflux-mediated resistance. Apart from these processes,*A. baumannii *has the ability to modify antibiotic target sites. For instance, mutations in ribosomal structures or penicillin-binding proteins can hinder the efficient binding of medicines. As a result, the medication's capacity to stop bacterial growth is diminished. The acquisition of resistance genes through horizontal gene transfer is another significant aspect. Through integrons, transposons, and plasmids, *A. baumannii *can acquire resistance genes from other bacteria. This capacity makes it possible for resistance to spread quickly in medical settings.

All things considered,* A. baumannii *is a highly adaptive and challenging-to-treat pathogen due to the combination of enzyme production, decreased permeability, active efflux, target modification, and gene acquisition. Choosing effective treatment plans and creating novel therapeutic approaches depend on an understanding of these mechanisms (Table 4) [5].

**Table 4:** Major Resistance Mechanisms in A. baumannii This table outlines the mechanisms of antimicrobial resistance in *Acinetobacter baumannii*, including enzyme-mediated carbapenem degradation, porin loss or modification, efflux pump activity, target site alterations, and acquisition of resistance genes. Each mechanism contributes to reduced antibiotic effectiveness and promotes the development and spread of multidrug-resistant (MDR) strains. The information is summarized from the referenced study. OXA: oxacillinase; MBL: metallo-β-lactamase

Mechanism	Description	Effect on antibiotics	References
Carbapenemase production	Enzymes (OXA-type, MBL) break down drugs	Inactivates carbapenems	[5]
Porin loss/modification	Reduced drug entry into the cell	Decreased antibiotic uptake	[5]
Efflux pumps	Actively expel antibiotics	Low intracellular drug concentration	[5]
Target site modification	Changes in binding sites	Reduced drug effectiveness	[5]
Gene acquisition	Transfer of resistance genes	Rapid spread of MDR	[5]

Biofilm formation and virulence in *Acinetobacter baumannii*

One of the most important adaptation strategies that helps *A. baumannii *survive in harsh conditions, especially in healthcare settings, is biofilm formation. In contrast to planktonic (free-living) bacteria, *A. baumannii *can form organized microbial communities embedded in a self-produced extracellular matrix by adhering to biotic and abiotic surfaces. Polysaccharides, proteins, and extracellular DNA make up the majority of this matrix, which together give the bacterial population protection and mechanical stability [6]. 

**Figure 2:**
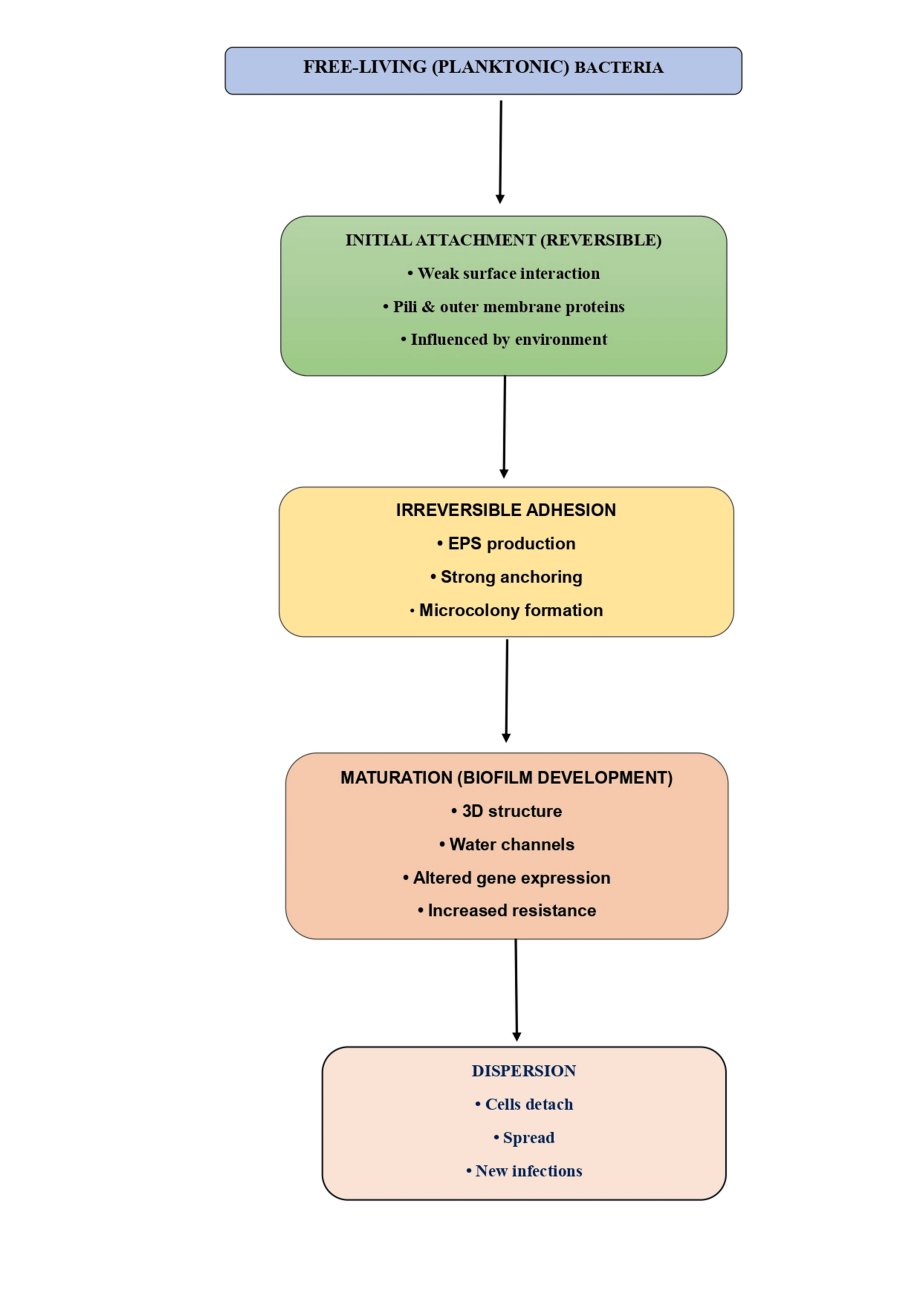
Stages of Biofilm Formation in A. baumannii. The figure was created manually using Microsoft PowerPoint 2021 for illustration purposes only and was not generated using AI software or AI-integrated features. The figure illustrates the sequential stages of biofilm development, beginning with free-living (planktonic) bacteria, followed by initial reversible attachment, irreversible adhesion with extracellular polymeric substance (EPS) production, maturation of the biofilm structure, and final dispersion leading to bacterial spread and new infections. 3D: three-dimensional

The clear correlation between recurrent infections and treatment failure makes biofilm formation clinically significant. *A. baumannii *commonly colonizes medical equipment in hospital settings, including endotracheal tubes, ventilators, urine catheters, and intravenous lines. These surfaces are perfect for the formation of biofilms, which makes it possible for bacteria to survive even after regular disinfection processes. Once formed, biofilms serve as a biochemical and physical barrier that protects bacteria from host immune systems and drastically lowers the penetration of antimicrobial agents [7]. The stages of biofilm formation in* A. baumannii* are shown in Figure 2.

Virulence factors contributing to biofilm formation

A number of significant virulence factors support A. baumannii's capacity to build biofilms:

Outer membrane protein A (OmpA) contributes to host cell damage and is essential for attachment to host cells and abiotic surfaces [6,8,9]. Biofilm-associated protein (Bap) is crucial for sustaining the stability and structure of biofilms throughout time. Pili and fimbriae promote early surface adhesion and colonization. Capsular polysaccharides offer defense against immunological reactions, antibiotics, and desiccation. These virulence factors boost the organism's capacity to produce severe and long-lasting infections in addition to promoting biofilm development [6,8,9].* *

Factors affecting the formation of biofilms

Numerous host-related and environmental factors influence biofilm formation, including nutrient availability, surface properties, pH, temperature, and antibiotic exposure. Nutrient-limited conditions may promote biofilm development as a survival strategy, while rough and hydrophobic surfaces facilitate bacterial attachment. Favorable environmental conditions and sub-inhibitory antibiotic concentrations may further enhance biofilm formation. Additionally, stress conditions, such as oxidative stress and antibiotic pressure, can increase bacterial survival and persistence within biofilms [8].

Prevalence and clinical relevance

One of the most significant virulence characteristics of *A. baumannii *is its capacity to form biofilms, which has been repeatedly shown in recent research to be present in a significant percentage of clinical isolates. Research indicates that most isolates, especially those from hospital environments, have moderate to strong biofilm-forming capacity, while the incidence of biofilm-producing bacteria varies by geography and healthcare setting [10]. This high incidence is particularly noticeable in ICUs, where invasive procedures and antibiotic use create selective pressure that helps these bacteria survive. Specifically, the development of biofilms is closely linked to both carbapenem resistance and multidrug resistance. CRAB isolates are more likely to form strong biofilms than susceptible strains, according to several investigations [6,10].

In clinical terms, biofilm-producing *A. baumannii* infections are more challenging to treat and frequently result in unfavorable patient outcomes. These infections are often chronic, necessitating long-term antibiotic treatment and raising healthcare expenses. VAP and catheter-related bloodstream infections are among the device-associated problems that form biofilms on medical devices, such as ventilators and catheters, are particularly problematic because they are ongoing sources of infection [7].

Biofilm production increases bacterial survival by preventing antibiotic penetration and promoting the exchange of resistance genes within the microbial population; this association is not purely accidental. Furthermore, biofilm-associated cells frequently display changed metabolic states, which lessen their susceptibility to antibiotics [8].

The presence of certain virulence genes, such as those encoding OmpA and Bap, supports the high frequency of biofilm formation at the molecular level. These genes increase the organism's capacity for pathogenicity by improving adhesion, structural integrity, and resistance [6,9]. 

All things considered, the prevalence of biofilm-producing*A. baumannii, *especially among MDR and CRAB strains, emphasizes the necessity of better infection control techniques and the devising of treatments aimed at biofilm-associated infections. Effective clinical care and lowering the prevalence of hospital-acquired infections depend on an understanding of this link (Table 5).

**Table 5:** Prevalence and Clinical Impact of Biofilm-Producing A. baumannii This table summarizes the prevalence and clinical impact of biofilm-producing *Acinetobacter baumannii *isolates. It highlights key aspects such as the high frequency of biofilm formation in hospital strains, its strong association with multidrug resistance and carbapenem resistance, and its role in device-associated infections. The table also outlines clinical consequences, including increased mortality, prolonged hospital stays, and enhanced pathogenicity mediated by virulence factors such as biofilm-associated protein (Bap) and outer membrane protein A (OmpA), based on the cited references. MDR: multidrug-resistant

Aspect	Observation	Clinical implication	References
Prevalence	The majority of isolates form biofilms	Common in hospital strains	[10]
Resistance link	Strong association with MDR and CRAB	Reduced antibiotic effectiveness	[6,8]
Device association	Biofilms on ventilators, catheters	Persistent infections	[7]
Clinical outcome	Increased mortality and hospital stay	Poor prognosis	[3,10]
Virulence genes	Bap, OmpA present	Enhanced pathogenicity	[6,9]

Relationship between biofilm formation and antimicrobial resistance

*Acinetobacter baumannii *has a complicated link between biofilm production and antibiotic resistance, which is crucial to the infection's persistence. Antibiotic resistance and biofilm formation frequently combine to increase bacterial survival under difficult circumstances, especially in hospital settings, rather than functioning as distinct mechanisms [11].

The fact that bacteria growing inside a biofilm behave considerably differently from free-living (planktonic) cells is one of the most important findings from recent research. While biofilm-associated cells dwell in a protected state with decreased metabolic activity, making them less receptive to antimicrobial drugs, planktonic cells are typically more metabolically active and consequently more susceptible to antibiotics. In clinical settings, therapy failure is largely caused by this variation alone.

Antibiotics cannot penetrate deeper layers of the bacterial community because biofilms operate as a physical barrier. Antibiotics are slowed down or prevented from reaching their target areas by the extracellular polymeric substance (EPS) matrix. Because of this, bacteria in the biofilm's inner layers are exposed to less-than-ideal medication concentrations, which may encourage the emergence of resistance [11].

Biofilms provide an environment that is conducive to genetic exchange in addition to providing physical protection. The close proximity of bacteria within biofilms makes it easier for resistance genes to spread via plasmids and other mobile genetic elements. Multidrug resistance in bacterial populations spreads quickly as a result of this process [11].

The existence of "persister cells" in biofilms is another crucial component. These bacteria are a tiny subpopulation that is extremely resistant to drugs and stays dormant. These cells can endure and then repopulate the biofilm, resulting in recurring infections, even after receiving antibiotic treatment. It is interesting to note that resistance and biofilm production are not always clearly related. Strong biofilm producers are generally more resistant, according to certain research. Highly resistant strains, however, may produce weaker biofilms, according to other research, suggesting a balance between resistance mechanisms and biofilm formation. Despite these differences, it is evident that biofilm production increases the organism's overall ability to survive. From a therapeutic perspective, this link has important ramifications. Higher dosages of antibiotics or combination therapy are frequently needed to eradicate biofilm-associated illnesses, and it is frequently challenging to do so without removing the contaminated equipment. This is especially important in ICUs, where the development of biofilms on medical equipment can lead to long-term and device-related illnesses (Table 6).

**Table 6:** Biofilm Versus Antimicrobial Resistance (Comparison) This table compares the characteristics of planktonic and biofilm-associated *Acinetobacter baumannii* cells, highlighting differences in metabolic activity, antibiotic penetration, gene transfer, presence of persister cells, and overall drug response. Biofilm cells exhibit reduced metabolic activity, limited antibiotic penetration due to the extracellular polymeric substance (EPS) barrier, increased gene exchange, and a higher prevalence of persister cells, all of which contribute to enhanced antimicrobial resistance, treatment failure, and recurrent infections, as supported by the cited references.

Feature	Planktonic cells	Biofilm cells	Clinical impact
Metabolic activity	High	Reduced	Lower drug susceptibility [11]
Antibiotic penetration	Easy	Limited (EPS barrier)	Treatment failure [11]
Gene transfer	Less frequent	Increased	Spread of resistance [11]
Persister cells	Rare	Common	Recurrence of infection [11]
Drug response	Sensitive	Highly resistant	Need for stronger therapy [11]

Role of biofilm in the pathogenesis of *Acinetobacter baumannii*

The ability of *A. baumannii* to endure harsh conditions and cause persistent infections, especially in hospitalized patients, is directly related to its pathophysiology [12-14]. *A. baumannii *uses a variety of mechanisms, including biofilm formation, surface adherence, immune evasion, and resistance characteristics, to cause disease, in contrast to many other bacteria that rely on a single virulence factor [15,16]. Initial colonization is one of the most crucial phases in the pathogenesis. The bacterium first uses structures such as pili and outer membrane proteins to stick to host tissues or medical equipment [17].

This connection is essential because it enables the bacteria to grow on surfaces such as wounds, indwelling medical devices, and respiratory epithelium. The bacteria start to grow and create biofilms once they are attached. The production of biofilms considerably increases*A. baumannii's* pathogenic potential. Bacteria are encased in an extracellular matrix that protects them from host immunological reactions and antibiotics within the biofilm. This makes it possible for the organism to endure for extended periods of time and evade removal, which can result in persistent or recurrent infections. Biofilm-associated infections are most prevalent in wound infections, VAP, and catheter-related infections [17,18].

Immune evasion is a key component of pathogenesis. Neutrophils and macrophages, two types of immune cells, have less access to microorganisms linked to biofilm. Furthermore, elements such as the outer membrane proteins and capsule aid the bacteria in avoiding complement-mediated death and phagocytosis. 

Laboratory studies and characterization of CRAB isolates

Accurate diagnosis, comprehension of resistance patterns, and guidance of suitable treatment approaches depend on laboratory characterization of CRAB. Phenotypic and molecular techniques are frequently employed in clinical microbiology labs to detect and examine these isolates [12]. Typically, normal culture methods and biochemical assays are used to identify* A. baumannii *first. On standard media, including conventional blood agar and MacConkey agar, the organism frequently forms smooth, opaque colonies. Another crucial component of characterization is biofilm detection. The potential of isolates to form biofilms is frequently evaluated using techniques such as the Congo red agar method, the tube method, and the tissue culture plate method, which is regarded as the gold standard.

These techniques aid in the correlation of resistance patterns and clinical consequences with biofilm development. The comprehension of CRAB isolates is further improved by molecular characterization. In hospital settings, genetic diversity, clonal connections, and transmission patterns are studied using techniques such as gene sequencing and typing approaches (such as multilocus sequence typing). Both epidemiological research and infection control benefit from this information [12]. Antimicrobial susceptibility testing (AST) is carried out to ascertain the isolate's resistance profile after it has been discovered. Automated systems or complex techniques such as matrix-assisted laser desorption ionization-time of flight (MALDI-TOF) mass spectrometry, which offer quick and accurate identification, can be used to obtain additional confirmation.

Following established protocols, the disk diffusion technique and broth microdilution are frequently employed. To categorize isolates as MDR or carbapenem-resistant, these assays aid in identifying resistance to carbapenems and other antibiotics [12].

In addition to conventional testing, certain techniques are employed to identify the production of carbapenemase. Carbapenemase enzymes can be detected using phenotypic assays such as the modified Hodge test or the carbapenem inactivation method. OXA-type carbapenemases are among the particular resistance genes that can be identified using more precise molecular methods, such as polymerase chain reaction (PCR) (Tables 7-8).

**Table 7:** Laboratory Methods for Diagnosis and Characterization of Acinetobacter baumannii (CRAB) This table outlines the laboratory methods used for the diagnosis and characterization of carbapenem-resistant *Acinetobacter baumannii *(CRAB), including conventional microbiological techniques, automated identification systems, antimicrobial susceptibility testing (AST), and advanced molecular approaches. It highlights the principles, examples, and clinical significance of each method, emphasizing their role in accurate identification, detection of resistance mechanisms, assessment of biofilm formation, and epidemiological tracking of CRAB isolates, as supported by the cited references. AST: antimicrobial susceptibility testing; MIC: minimum inhibitory concentration; CRAB: carbapenem-resistant *Acinetobacter baumannii;* ESBL: extended-spectrum β-lactamase; MBL: metallo-β-lactamase

Step	Method	Principle/description	Example/test	Purpose	Clinical significance	References
1	Gram staining	Gram-negative coccobacilli, short rods, often seen in pairs	Gram stain from the clinical sample	Preliminary identification	Rapid initial clue in suspected infections	[12]
2	Culture and colony morphology	Growth on routine media, non-lactose fermenting	Blood agar (smooth, opaque colonies), MacConkey agar (pale colonies)	Isolation of the organism	Essential for further testing	[12]
3	Motility test	Non-motile organism	Hanging drop/semi-solid agar	Differentiation from motile gram-negative bacilli	Helps in identification	[12]
4	Biochemical tests	Basic metabolic characteristics	Oxidase (negative), catalase (positive), OF test (oxidative)	Confirmation of the genus	Distinguishes from other non-fermenters	[12]
5	Automated identification systems	Uses biochemical and proteomic profiling	VITEK 2, MALDI-TOF MS	Rapid and accurate identification	High precision, widely used in clinical labs	[12]
6	Antimicrobial susceptibility testing (AST)	Determines the antibiotic resistance pattern	Disk diffusion, broth microdilution (MIC) as per CLSI	Detect MDR/CRAB strains	Guides appropriate therapy	[12,13]
7	Carbapenem resistance screening	Detection of resistance to carbapenems	Imipenem/meropenem disk testing	Screening of CRAB	Early detection of resistant isolates	[5,12]
8	Phenotypic carbapenemase detection	Detects enzyme activity	Modified Hodge test (MHT), carbapenem inactivation method (CIM), EDTA synergy test	Confirm carbapenemase production	Important for resistance mechanism identification	[5,12]
9	Biofilm detection assays	Detects the ability to form a biofilm	Tissue culture plate (TCP) method (gold standard), tube method, Congo red agar	Assess virulence factors	Predicts chronic and device-associated infections	[6,7,12]
10	Molecular detection (PCR)	Detects resistance genes	PCR for OXA-type genes (blaOXA-23, blaOXA-51), MBL genes	Genetic confirmation	Highly specific and sensitive	[5,12]
11	Genotyping methods	Determines strain relatedness	MLST (multilocus sequence typing), PFGE	Epidemiological tracking	Helps in outbreak investigation	[12]

**Table 8:** Antimicrobial Susceptibility Testing (AST) and Resistance Pattern of Acinetobacter baumannii (CRAB) This table summarizes the antimicrobial susceptibility patterns of carbapenem-resistant *Acinetobacter baumannii* (CRAB) across different antibiotic classes, along with the corresponding resistance mechanisms and clinical interpretations. It highlights the widespread resistance to multiple antibiotic groups, including β-lactams, cephalosporins, carbapenems, and fluoroquinolones, while noting limited susceptibility to agents such as tigecycline and colistin. The table also emphasizes the role of combination therapy in improving treatment outcomes in severe infections. The information is based on antimicrobial susceptibility testing (AST) results and supported by the cited references.

Antibiotic class	Antibiotic	Testing method	Observed resistance pattern in CRAB	Mechanism of resistance	Clinical interpretation	References
Penicillin + β-lactamase inhibitors	Piperacillin-tazobactam	Disk diffusion/MIC	High resistance	β-lactamase production	Limited clinical use	[5,12,13]
Cephalosporins	Ceftazidime, cefepime	Disk diffusion/MIC	Very high resistance	ESBL production, porin loss	Not preferred	[5,12]
Carbapenems	Imipenem, meropenem	Disk diffusion/MIC	Resistant (CRAB definition)	OXA-type carbapenemases, MBL	Key indicator of CRAB	[5,12,13]
Aminoglycosides	Amikacin, gentamicin	Disk diffusion/MIC	Variable resistance	Enzymatic modification, efflux pumps	Sometimes useful in combination	[5,13]
Fluoroquinolones	Ciprofloxacin, levofloxacin	Disk diffusion/MIC	High resistance	DNA gyrase mutation, efflux	Limited use	[5]
Tetracyclines	Tigecycline	MIC (preferred)	Moderate susceptibility	Efflux pumps	Used as last-line therapy	[13]
Polymyxins	Colistin (polymyxin E)	Broth microdilution (gold standard)	Usually sensitive (emerging resistance reported)	Membrane modification	Drug of choice (last resort)	[13]
Sulfonamides	Trimethoprim-sulfamethoxazole	Disk diffusion	High resistance	Target modification	Rarely used	[5]
Combination therapy	Colistin + meropenem/tigecycline	MIC-based synergy testing	Improved activity	Synergistic effect	Recommended in severe infections	[13]

Treatment and control strategies for CRAB infections

Due to the organism's capacity to form biofilms and the lack of effective antibiotic alternatives, managing infections caused by CRAB continues to be a significant therapeutic issue. Several envelope-associated determinants contribute to antimicrobial resistance, virulence, persistence, and surface variability in *A. baumannii. *Antimicrobial medication, supportive care, and stringent infection control measures are necessary components of treatment plans, which are frequently complicated [13].

Traditional Antibiotic Treatment

Antibiotics such as aminoglycosides, tigecycline, and colistin have traditionally been employed as a last resort to treat CRAB infections. Colistin is generally regarded as one of the most efficient of these, although its use is linked to serious damage, especially nephrotoxicity. Although its efficacy in treating bloodstream infections is limited, tigecycline is an additional alternative, particularly for soft tissue infections [13]. Monotherapy is frequently inadequate due to growing resistance. Combination therapy has become more significant as a result. Combining medications such as colistin with tigecycline or meropenem may improve antibacterial activity and lower the risk of treatment failure [13].

Stewardship of Antibiotics

Controlling the spread of CRAB requires the prudent use of antibiotics. The development of resistance is greatly aided by the overuse and abuse of broad-spectrum antibiotics. By ensuring proper therapy selection, dosage, and duration, antibiotic stewardship programs help lessen the selective pressure on bacteria [13].

Anti-Biofilm Therapeutic Approaches

Because standard medications have a hard time penetrating the protective matrix, biofilm-associated illnesses are especially challenging to treat. As a result, focusing on biofilm formation has grown in importance as a field of study. Agents that damage the extracellular matrix, prevent bacterial adherence, or obstruct quorum-sensing processes are examples of anti-biofilm tactics. By weakening the biofilm structure, these methods seek to increase the susceptibility of bacteria to antibiotics [14].

Additionally, the potential of several chemicals to stop biofilm growth on medical equipment has been investigated. Infection rates in healthcare settings can be decreased by applying antimicrobial coatings to surfaces or by utilizing materials that are resistant to bacterial adhesion [14].

*Emerging and Novel Therapies* The goal of recent research has been to create novel therapeutic approaches to combat resistance (Table 9). Some of these are:

Nanoparticle-based treatments: Antimicrobial medicines can be directly delivered to bacterial cells using nanoparticles, which can break through biofilms. Phage therapy: Bacteriophages attack and kill bacterial cells, particularly those found in biofilms. Antimicrobial peptides: These peptides exhibit activity against resistant strains of bacteria by rupturing their membranes. These methods provide viable substitutes, particularly when traditional antibiotics are ineffective [15].

**Table 9:** Advanced Therapies for Biofilms in CRAB This table summarizes advanced and emerging therapeutic approaches targeting biofilm-associated infections in Acinetobacter baumannii. It outlines various strategies, including nanoparticle-based treatments, anti-biofilm agents, enzymatic therapies, and physical methods, along with their mechanisms of action, potential benefits, and current limitations. These approaches aim to enhance antibiotic efficacy by disrupting biofilm structure and improving drug penetration, as supported by the cited references.

Therapy type	Mechanism	Benefit	Limitation	References
Nanoparticles	Penetrate biofilm, disrupt cells	High effectiveness	Still under study	[9,16]
Anti-biofilm agents	Inhibit adhesion and EPS	Improve antibiotic action	Limited clinical use	[9,16]
Enzymatic therapy	Break down the biofilm matrix	Better drug penetration	Experimental stage	[9,16]
Physical methods	Disrupt biofilm structure	Non-antibiotic approach	Not widely available	[9,16]

*Prevention and Control of Infections* Strict commitment to infection prevention procedures is necessary for the effective control of CRAB infections. Important preventive measures include hand hygiene, sterilization and disinfection of medical equipment, isolation of infected patients, and surveillance of hospital-acquired infections. Regular monitoring and adherence to infection control protocols are essential to prevent outbreaks, particularly in ICUs, where the risk is the highest. In addition, appropriate interpretation of antimicrobial susceptibility testing (AST) patterns is important for guiding effective infection management and therapeutic decisions in CRAB infections (Table 10).

**Table 10:** Interpretation of AST Pattern This table defines key antimicrobial resistance patterns observed in *Acinetobacter baumannii,* including multidrug-resistant (MDR), extensively drug-resistant (XDR), pan drug-resistant (PDR), and carbapenem-resistant A. baumannii (CRAB). It outlines their respective definitions and clinical implications, highlighting the progressive limitation of treatment options and the increasing severity of clinical outcomes associated with higher levels of resistance, as supported by the cited references. Note: AST plays a crucial role in guiding therapy, especially in CRAB, where resistance is widespread. Accurate interpretation using CLSI/EUCAST guidelines is essential for optimal patient management. CRAB, carbapenem-resistant *Acinetobacter baumannii; *CLSI/EUCAST: Clinical and Laboratory Standards Institute/European Committee on Antimicrobial Susceptibility Testing

Pattern	Definition	Clinical meaning	References
MDR (multidrug-resistant)	Resistant to ≥ three antibiotic classes	Limited treatment options	[5,13]
XDR (extensively drug-resistant)	Resistant to all except 1-2 classes	Very few treatment options	[5,13]
PDR (pan drug-resistant)	Resistant to all available antibiotics	Critical condition	[5,13]
CRAB	Resistant to carbapenems	High mortality risk	[1,5]

## Conclusions

In hospital settings around the world, CRAB has become a serious concern, especially in ICUs where infection rates and related mortality are still high. It is one of the most difficult infections to successfully treat due to its extraordinary capacity to endure harsh environments and quickly develop resistance mechanisms. Its ability to produce biofilms is a major component in the persistence of CRAB infections. By restricting drug penetration and encouraging resistance mechanisms, biofilm development not only increases bacterial survival but also dramatically lowers the efficacy of antimicrobial therapy. An important factor in persistent and recurring infections is the complex connection between biofilm formation and antibiotic resistance. Due to toxicity, developing resistance, and the protective properties of biofilms, treatment results are frequently less than ideal, even if last-resort antibiotics such as colistin and tigecycline are still utilized. To increase treatment efficacy, new strategies, such as anti-biofilm tactics and cutting-edge therapeutic techniques, are becoming increasingly popular. Early detection, proper antimicrobial medication, strict infection control procedures, and the establishment of antibiotic stewardship programs are necessary for the effective management of CRAB infections. Additionally, greater research into biofilm biology and resistance mechanisms is necessary to create more focused and potent treatments. In summary, CRAB poses a significant and dynamic threat to world health. Treatment and control are made much more difficult by the combination of biofilm formation and antibiotic resistance. Coordinated efforts in clinical treatment, research, and infection prevention are needed to address this problem and lessen its negative effects on patient outcomes.
